# Layering and packing in confined colloidal suspensions

**DOI:** 10.1039/d2sm00412g

**Published:** 2022-06-07

**Authors:** Alejandro Villada-Balbuena, Gerhard Jung, Angel B. Zuccolotto-Bernez, Thomas Franosch, Stefan U. Egelhaaf

**Affiliations:** Condensed Matter Physics Laboratory, Heinrich Heine University Universitätsstraße 1 40225 Düsseldorf Germany stefan.egelhaaf@uni-duesseldorf.de; Institut für Theoretische Physik, Universität Innsbruck Technikerstraße 21A 6020 Innsbruck Austria; Laboratoire Charles Coulomb (L2C), Université de Montpellier, CNRS 34095 Montpellier France

## Abstract

Confinement modifies the properties of a fluid. The particle density is no longer uniform but depends on the distance from the walls; parallel to the walls, layers with different particle densities form. This affects the particle packing in the layers. We investigated colloidal fluids with volume fractions between 0.19 and 0.32 confined between rough walls. The particle–particle interactions were dominated by hard-sphere interactions but also contained some electrostatic interactions. The particle locations were determined using confocal microscopy and served to calculate the density profile, radial distribution function, anisotropic and generalized structure factors but also to characterize the arrangement of the wall particles leading to the roughness of the walls. The experiments are complemented by molecular dynamics simulations and fundamental-measure theory. While the particle arrangements are mainly controlled by hard-core interactions, electrostatic interactions become more important as the volume fraction decreases. Furthermore, the structure of the rough walls was varied and found to have a significant effect on the fluid structure. An appropriate representation of the rough walls in the simulations is thus crucial to successfully mimic the experiments.

## Abstract 2

Confinement modifies the properties of a fluid. We investigated the inhomogeneous density profiles and the anisotropic packing of colloidal fluids confined between parallel rough walls using experiments, computer simulations and theory.

## Graphical abstract

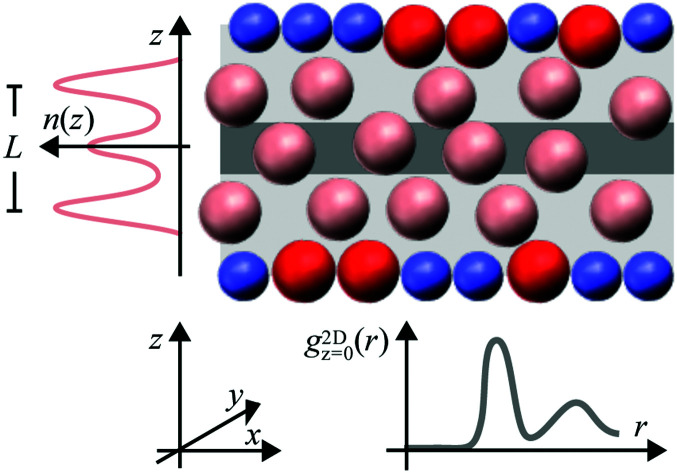

## 1 Introduction

The properties of fluids are modified by physical confinement. Confinement changes, for example, the structure and phase behaviour,^1–12^ dynamics and viscoelasticity,^12–16^ and glass transition.^17–24^ It affects atomic, molecular, as well as colloidal fluids. Correspondingly, confinement is relevant for many areas, ranging from physics, chemistry, biology, and medicine to environmental and material sciences as well as technology. Applications include, *e.g.*, nanopatterning and nanofabrication, coatings, tribology including lubrication and friction, micro- and nanofluidics, molecular sieving, liquids in porous media or the interiors of cells, blood components in vessels and channels in biological membranes.

A bulk fluid is isotropic and disordered except for a local structure characterized by short-ranged density variations.^25^ A wall, however, leads to wetting and results in a nonuniform density profile with layers parallel to the wall.^26^ The density profile perpendicular to the wall hence shows modulations. The length scale of the modulations corresponds to the particle size. The modulations extend from the wall into the fluid over a range governed by the position correlations in the fluid structure. Confinement between two walls similarly breaks translational symmetry. It leads to anisotropic ordering with distinct layers parallel to the walls and transport properties that differ from those found in bulk.^5–8,12,14,27–36^ The incompatibility between the isotropic structure of bulk fluids and the anisotropic layering imposed by confinement results in density variations that are particularly pronounced when both effects are commensurate and hence mutually reinforce each other.^27^ The packing of spheres in layers is particularly efficient, and hence the layering most pronounced, for wall separations slightly less than an integer multiple of the particle diameter. Upon increasing the wall separation, an alternating sequence of highly anisotropic layering and a more isotropic local order is observed.

**Fig. 1:**
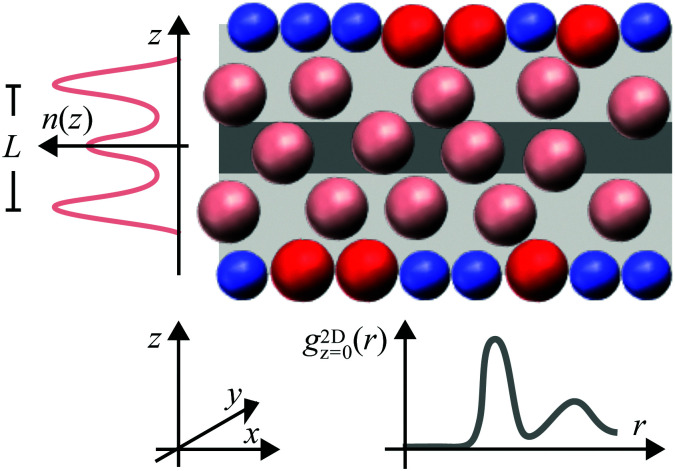
Schematic representation of a colloidal fluid confined between rough walls (not to scale). The rough walls are formed by fixed wall particles (blue) and immobile fluid particles (red). The layering of the mobile fluid particles (pink) parallel to the walls is characterized by the density profile *n*(*z*) where the *z* direction is defined perpendicular to the walls. The packing within the layers, *i.e.* parallel to the walls in *x* and *y* direction, is described by the two-dimensional radial distribution function *g*^2D^_*z*_(*r*), where particles in a slab centred around *z* (here *z* = 0, dark grey region) are considered and *r* = (*x*^2^ + *y*^2^)^1/2^ is the distance parallel to the walls. The confinement length *L* is defined as the separation between the outermost maxima of *n*(*z*).

The density modulations perpendicular to the walls can be quantified by the density profile *n*(*z*), where the *z* direction is defined perpendicular to the walls with *z* = 0 in the centre of the slit (Fig. 1). The modulations extend throughout the whole slit if the wall separation is small, up to a few times the particle size, and the particle concentration is high enough, such that the particle–particle and particle–wall interactions are important. Correspondingly, the packing within the layers depends on the wall separation and the distance to the walls. In the layers parallel to the walls, a pronounced local order with significant density modulations can develop^28^ and, in concentrated systems, a fluid–crystal transition can occur.^2^ The packing of particles parallel to the walls can be characterized by the distribution of particles around a specific particle and is quantified by the two-dimensional positions of particles in a slab parallel to the walls, the two-dimensional radial distribution function (2D-RDF) *g*^2D^_*z*_(*r*) (Fig. 1). The radial distribution functions can be strongly anisotropic and show a pronounced dependence on the position within the slit, *z*.^6,29,30^ Both, layering and packing, have together been described by the local density correlation function^8,27–31^ or the structure factor *S*(***q***).^8,18–20,29,30,37^ Due to the symmetry of the confined fluid, the fluid structure can be fully characterized in dependence of two parameters, the distance to the central plane, *z*, and the particle–particle distance within a plane parallel to the walls, *r* = (*x*^2^ + *y*^2^)^1/2^ (Fig. 1). Similarly, the structure factor is studied in dependence of the magnitudes of the wavevectors parallel, *q*_‖_, and perpendicular, *q*_⊥_, to the walls.

Particles with a variety of shapes and differently-shaped cavities have been investigated.^9–11,13^ However, the simplest possible system consists of hard-spheres confined by hard walls. This situation has been investigated using computer simulations, density-functional theory and integral-equation theory^5,6,12,27,31,32,38^ and experiments based on force measurements,^39–42^ scattering methods^6,29,30,33–35,43–50^ as well as conventional and confocal microscopy.^51–58^ In addition, the effects of charges on the particles and walls have been studied, for example their effects on the phase behaviour^59^ and packing.^33–35,45,48,49^ In both cases, hard and charged walls, the walls are in general isotropic in the plane of the wall. Randomly structured, *e.g.* patchy or rough, walls have been less studied and, if so, the emphasis has been on the particle dynamics in confinement^60–69^ or close to individual rough walls.^22,70,71^

Using experiments, simulations and theoretical calculations, we investigate the effects of confinement by rough walls on moderately dense fluids. We consider spherical colloids. The particle–particle as well as the particle–wall interactions were dominated by hard-core interactions but also contained some electrostatic contribution. The amplitude of the roughness, that is the typical variation in the wall relief, has been chosen small enough to still allow for layering and for an unambiguous definition of the slit width, but large enough to have an effect on the arrangement of the particles. This implies an amplitude of the roughness smaller than but similar to the particle diameter. Roughness is introduced by decorating the walls with particles that are similar to the particles forming the fluid.

The experimental system consisted of micron-sized poly(methyl methacrylate) (PMMA) spheres with, mainly, hard-sphere-like interactions but also a small amount of residual charges. Their volume fraction covered a range 0.19 ≲ *ϕ* ≲ 0.32. They are confined between two walls formed by randomly-arranged fixed particles and immobile fluid particles. The wall separation is quantified by the confinement length *L* corresponding to the separation between the outermost maxima of the density profile *n*(*z*) (Fig. 1). The confinement length *L* has been varied. The colloidal size allowed us to image the samples using confocal microscopy. This yields the location of each particle and thus permits for a quantitative analysis on an individual particle level. Thus, a detailed characterization of the confined fluid as well as the particles forming the rough walls is possible. The focus is on the density profile *n*(*z*), the two-dimensional radial distribution function *g*^2D^_*z*_(*r*) and the anisotropic, *S*(*q*_‖_,*q*_⊥_), as well as the generalized, *S*_*μν*_(*q*_‖_), structure factors. Although the samples are investigated by confocal microscopy and hence real space information on each single particle is obtained, also the structure factors are considered because they provide complementary and detailed information on the particle arrangement and allow for a comparison with scattering experiments and theoretical predictions.

The experimental systems are mimicked in molecular dynamics simulations. The simulation results quantitatively agree with the experimental findings. The experimental findings and simulation results are moreover compared to predictions from fundamental-measure theory for hard-spheres confined by flat soft walls. Thus, the experimental findings can be compared with simulation results as well as theoretical predictions. The main aims are to quantify the effects of a moderate particle charge and of the roughness of the walls and to faithfully implement the roughness of the walls in the simulations to be able to mimic the experiments. While the fluid structure is dominated by excluded-volume effects, *i.e.* hard-core interactions, the electrostatic interactions are found to become more important at low volume fractions. Moreover, the effects of the wall roughness are investigated by simulations employing different rough and flat walls. Our findings indicate that the structure of the fluid significantly depends on the details of the rough walls. They also show that a detailed determination of the roughness, as accessible through confocal microscopy, as well as a subsequent realistic representation of the roughness of the walls in the simulations is crucial to achieve quantitative agreement with the experimental findings.

## 2 Materials and methods

### 2.1 Sample preparation and imaging

#### 2.1.1 Samples

The samples contained poly(methyl methacrylate) (PMMA) spheres that were locked, stabilized with poly(12-hydroxy-stearic acid) (PHSA)^72^ and fluorescently labelled with rhodamine B that was monomerized with methyl methacrylate. After the synthesis, the particles were kept in decalin, *i.e.* a mixture of *cis* and *trans*-decahydronaphthalene (decalin, purity >98%, Alfa Aesar). Decalin was exchanged for *cis*-decahydronaphthalene (*cis*-decalin, purity >98%, TCI) by sedimenting the particles through centrifugation and subsequently exchanging the supernatant. This procedure was repeated three times. Afterwards, the samples were repeatedly centrifuged and the supernatant exchanged for a mixture of *cis*-decalin and cyclohexyl bromide (CHB6, purity >98%, TCI).^73^ The mixing ratio of *cis*-decalin and CHB6 was adjusted to match the densities of the solvent mixture and the particles (resulting for the present particles in a volume ratio of *cis*-decalin to CHB6 of 0.3755) to avoid sedimentation or creaming of the particles. This resulted in a refractive index of the solvent mixture that was close to the one of the particles, rendering the suspension sufficiently transparent to allow for confocal microscopy. In CHB6, the particles acquire a charge,^73,74^ especially if they are locked.^75^ In addition, rhodamine B charges the particles.^76^ In order to screen the charges, 4 mM tetrabutylammonium chloride (TBAC) was added.^73^ Samples with volume fractions *ϕ* = 0.19, 0.20, 0.28 and 0.32 were prepared assuming the sediment is random close packed with a volume fraction *ϕ*_RCP_ = 0.65.^77^ These volume fractions were confirmed by confocal microscopy (Section 2.4.4).

The particles have a mean diameter *σ*_p_ = 1.85 μm as determined by confocal differential dynamic microscopy.^78^ Within the expected uncertainty, this diameter is consistent with results from static light-scattering experiments^79^ that yielded a mean diameter of 1.80 μm and a polydispersity *δ*_p_ = 4.8%.

#### 2.1.2 Sample cells

**Fig. 2:**
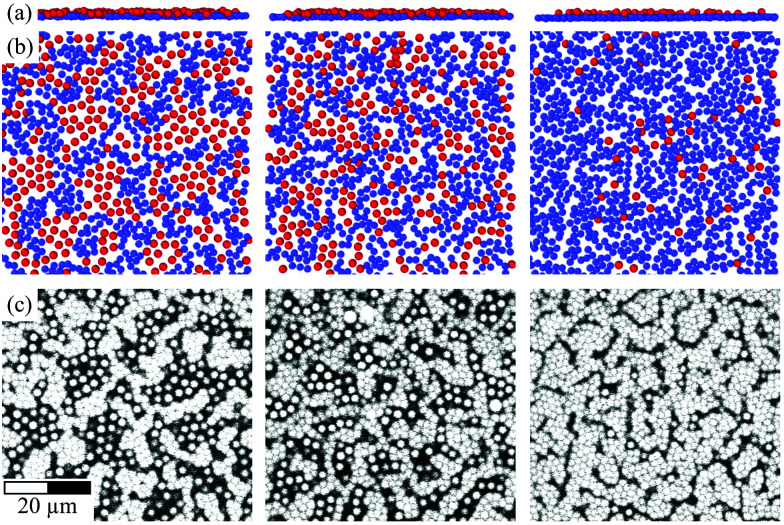
Layer of fixed wall particles (blue) and immobile fluid particles (red) schematically represented in side view (a) and bottom view (b), where the images were created using Ovito,^80^ as well as the corresponding confocal microscopy images (c). The variations in the wall coverage are caused by slight differences in the preparation procedure but not by the volume fraction of the sample, here *ϕ* = 0.32, or the confinement length *L*, here *L* = 1.9*σ*_p_ (left), 2.4*σ*_p_ (middle), 4.4*σ*_p_ (right). The scale bar represents 20 μm.

The samples were investigated in narrow cells consisting of two cover glasses coated with PMMA particles identical to the fluid particles and are initially dispersed in the identical solvent mixture (Section 2.1.1). The cells were prepared as follows. Two rectangular borosilicate cover glasses with areas 18 × 18 mm^2^ and 24 × 50 mm^2^ and a thickness of approximately 220 μm (number #2, Marienfeld) were washed with isopropanol. On each cover glass, a cylindrical glass tube with an inner diameter of 1.28 cm was placed. The tube was filled with 26 μl of a suspension containing PMMA particles with a volume fraction *ϕ* = 0.01. Then decalin was added to introduce a density mismatch and hence particle sedimentation. Subsequently, the solvent was evaporated by placing the cover glasses with the cylindrical glass tube in a vacuum oven at a temperature of 70 °C and a pressure of 0.2 mbar for 48 h. This procedure resulted in a circular amorphous monolayer of PMMA particles on the cover glasses. To create a spacer, 4 μl of a suspension containing PMMA particles with a volume fraction *ϕ* = 0.40 was deposited on the smaller cover glass next to the circular layer of PMMA particles and along one edge. On the larger cover glass it was deposited at the corresponding position, *i.e.* next to the circular layer of PMMA particles but further from the edge. The suspension was left to dry in the vacuum oven under the same conditions as before for 48 h. This resulted in spacers with an area of 18 × 3 mm^2^ and a height corresponding to about 10 particle layers. Once the solvent was evaporated and hence the particles dried, the coated cover glasses were kept at a temperature of 180 °C and a pressure of 1 atm for 6 h. This partially melted the particles and fixed them to each other and to the cover glass. During this process, the particles shrank to a diameter *σ*^w^_p_ ≈ 1.65 μm, as estimated by confocal microscopy, and acquired some additional charge due to the extended handling, including a prolonged exposure to air. Minute variations in the procedure followed to coat the cover slips can change the coverage of the walls and the arrangement of the particles, especially the homogeneity of the monolayer of particles (Fig. 2c). This resulted in some variation of the wall roughness between different sample cells and different positions in the same sample cell.

Finally, approximately 4 μl of sample was placed on the circular monolayer of the larger cover glass and covered by the smaller cover glass with its circular monolayer and spacer facing the layer and spacer of the larger cover glass. Once the sample had spread throughout the whole volume, the edges were sealed with glue (All Purpose Adhesive Super, UHU). The glue was left to dry for 48 h. Thus the sample was contained in a narrow, wedge-shaped slit with a maximum height of 20 to 30 μm. This implies a very small inclination angle of less than 0.1°. Thus different wall separations could be investigated using the same sample cell while the wall separation was almost constant in one particular field of view. The difference in slit height that occurs in one direction across the field of view due to the inclination angle depends on the imaging conditions (Section 2.1.3) but was always smaller than 0.1 μm and hence more than an order of magnitude smaller than the mean particle diameter. The samples were kept and investigated at a temperature between 19 °C and 20 °C.

#### 2.1.3 Confocal microscopy and particle tracking

The samples were imaged with a confocal scanning unit (A1R-MP, Nikon) mounted on an inverted microscope (Ti-U, Nikon) and using a solid-state laser with a wavelength of 561 nm. Three oil immersion objectives were used with different magnifications (and zoom settings) and numerical apertures that yielded different pixel pitches; 40× (zoom 3), NA = 1.30 (Plan Fluor, Nikon) resulting in 0.207 μm px^−1^; 60× (zoom 2), NA = 1.40 (Plan Apo VC, Nikon) resulting in 0.210 μm px^−1^; 100× (zoom 2), NA = 1.40 (Plan Apo VC, Nikon) resulting in 0.124 μm px^−1^.

At different positions in the samples corresponding to different wall separations, image stacks were obtained. The stacks consisted of individual slices with 512 × 512 pixels. The stacks covered the whole slit and were collected from the bottom to the top of the slit in steps of 0.25 μm. To scan one stack took 4 to 6 s depending on the wall separation. For each position between 3000 (large widths) and 5000 (small widths) stacks were recorded resulting in a total measurement time per position of about 5 h.

From the stacks, particle locations were extracted using standard algorithms implemented using interactive data language (IDL).^81^ To detect the fluid particles, a reconstruction diameter between 1.8 and 2.0 μm was used whereas a reliable detection of the fixed wall particles required to reduce the reconstruction diameter to 0.6 μm because of the loss of dye in these particles during the preparation of the sample cell. The obtained particle locations were analyzed as described below (Section 2.4) using Python routines.^82^

### 2.2 Molecular dynamics simulations

#### 2.2.1 Simulation algorithm and interaction potential

Molecular dynamics simulations of a confined fluid consisting of particles with hard-sphere and electrostatic interactions were performed. The hard-sphere interactions were modelled as previously suggested.^83^ Two spheres, *i* and *j*, separated by *r*_*ij*_ interact *via* a purely repulsive Lennard-Jones-like (LJ) potential1
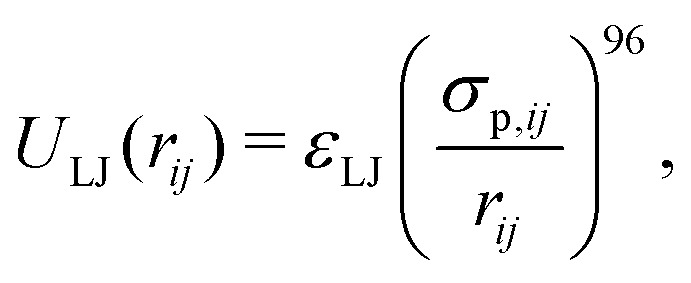
with the effective sphere diameter *σ*_p,*ij*_ = (*σ*_p,*i*_ + *σ*_p,*j*_)/2, the LJ energy scale *ε*_LJ_ = 1000*ε* and the energy unit of the simulations, *ε*. It was checked that the specific form and parameters of the LJ potential do not affect the results presented in this work. To account for the residual particle charges, in addition a Yukawa-like repulsion was included2
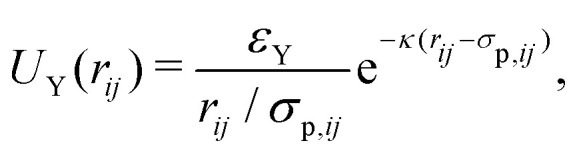
with the inverse Debye length *κ* and the Yukawa-energy scale *ε*_Y_.

The simulation units are given by the energy scale *ε*, the charge *e*, the length scale *σ* and the time scale *τ*. They can be translated into SI units by comparison to the experimental parameters. The unit of energy *ε* is given by the thermal energy *k*_B_*T* = T*ε with T* = 1 in the simulations. Since the experiments were performed at room temperature, the energy scale *ε* = 4.1 × 10^−21^ J. The unit of charge *e* can directly be identified with the elementary charge *e* = 1.602 × 10^−19^ C. The unit of length *σ* is defined by *σ* = *σ*_p_ = 1.85 μm. The unit of time, *τ*, was not mapped since only static properties are of interest here.

The system is integrated in the canonical ensemble using a Langevin thermostat to maintain constant temperature (relaxation time scale *τ*_L_ = *τ*). Since we aim to extract static properties only, we have chosen this approach although it does not include hydrodynamics. The time step was set to Δ*t* = 10^−4^*τ* and the particle mass to *m* = 0.25*ετ*^2^*σ*^−2^. The size of the simulated system corresponds to the experimental observation volume; approximately 30 × 30 particles parallel to the walls and 3 to 6 particle layers across the slit where periodic boundary conditions were applied in both directions parallel to the walls. This results in approximately 2700 to 5600 particles depending on the confinement length *L*. The systems were equilibrated before measurements were started.

#### 2.2.2 Rough walls

In the experiments, the walls were covered with a monolayer of fixed particles that, during the preparation procedure, shrank and acquired additional charges and hence were smaller and higher charged than the fluid particles (Section 2.1.2). Confocal microscopy revealed that these particles covered most of the wall but some uncovered regions remained (Fig. 2c). The uncovered regions were occupied by fluid particles that hardly moved within these regions and only very few were observed to leave these regions. These fluid particles were thus considered to be immobile. Thus the rough walls were formed by two kinds of particles; fixed wall particles and immobile fluid particles. The presence of two species with slightly different sizes and significantly different numbers of charges introduces some heterogeneity and hence an additional roughness with a slightly larger length scale depending on the number ratio of the two species. Small variations in the preparation procedure affect this ratio resulting in walls with different roughnesses (Fig. 2c). (Note that, in contrast, the roughness is independent of, *e.g.*, the confinement length *L* and volume fraction *ϕ* although the roughness is different for different *L* and *ϕ* due to slight differences in the preparation of the walls.) To mimic this behaviour in the simulations, the walls were covered by two types of particles; fixed wall particles and immobile fluid particles (see also the beginning of Section 3). The immobile fluid particles had the same properties, especially size and interactions, as the fluid particles but were immobile, which was modelled by an infinite mass. The fixed wall particles were smaller, *σ*^w^_p_ < *σ*_p_, and, due to the extensive handling, are expected to have a higher charge than the fluid particles, *ε*^w^_Y_ > *ε*_Y_ (Section 2.1.2) and were also immobile, again modelled by an infinite mass. The sizes of all particles were drawn from a Gaussian distribution with a polydispersity *δ*_p_ = 4.8% to mimic the experiments (Section 2.1.1).

The simulations were initialized with configurations taken from confocal microscopy images that contained all particles, *i.e.* mobile and immobile fluid particles and fixed wall particles. The different kinds of particles were classified based on the experimental density profiles *n*(*z*) containing mobile and, for this analysis of the wall particles, also immobile fluid particles and fixed wall particles (Section 2.4.1). Particles were identified as fixed wall particles if they are located within a distance *L*_w_ = 0.35*σ*_p_ from the outermost maxima of the density profile. Immobile fluid particles are larger than fixed wall particles and hence are expected further from the outermost maxima but they are part of the rough wall and hence are closer to the outermost maxima than the fluid particles. Thus, particles within a range *L*_w_ = 0.35*σ*_p_ to *L*_im_ = 0.81*σ*_p_ from the outermost maxima are considered immobile fluid particles if, in addition, they do not overlap with fixed wall particles. All other particles are defined as (mobile) fluid particles. (Note that the monolayers next to the walls, which contain only fixed wall and immobile fluid particles, are only taken into account here but not in the further analysis; Section 2.4.) The results are not significantly altered if the values of *L*_w_ and *L*_im_ are changed within reason. This procedure resulted in a full coverage of the walls (Fig. 2a and b). It also takes into account experimental variations, such as the exact coverage with fixed wall particles and their arrangement (Fig. 2c). This is important because the coverage and arrangement depend on minute details of the experimental preparation procedure followed to coat the cover slips (Section 2.1.2) and are hence difficult to precisely and consistently reproduce in the experiments. The coverage and arrangement do not directly but implicitly depend on the parameters varied in this study, such as the wall separation and the volume fraction of the sample.

#### 2.2.3 Parametrization of the interaction potential

The values of the parameters characterizing the Yukawa-like repulsion, namely the inverse Debye length *κ* and the Yukawa-energy scale *ε*_Y_ (eqn (2)), were chosen such that the simulation results match the experimental observations.

The values of the inverse Debye length were determined to *κ* ≈ 19*σ*_p_^−1^ = 10 μm^−1^ with some variations between the samples (Table 1). The variations are attributed to, *e.g.*, the low solubility of the salt and solvent evaporation during sample preparation which was difficult to avoid due to the relatively large surface area of the sample during its spreading on the cover slip. The magnitude of the Debye length *κ*^−1^ ≈ 100 nm is within the range previously reported for similar systems.^73,83^

**Table 1:** Parameters characterizing the Yukawa-like repulsive potential used in the simulations (eqn (2)) and values chosen to match the experimental observations; volume fraction of the sample *ϕ*, inverse Debye length *κ* in units of the inverse mean particle diameter 1/*σ*_p_ = 0.541 μm^−1^, Yukawa-energy scales of the mobile and immobile fluid particles, *ε*_Y_, and the fixed wall particles, *ε*^w^_Y_, in units of the energy scale *ε* = 4.1 × 10^−21^ J, *i.e.* the thermal energy, and number of elementary charges of the mobile and immobile fluid particles, *Z*, and the fixed wall particles *Z*^w^

*ϕ*	*κσ*_p_	*ε*_Y_/*ε*	*ε* ^w^_Y_/*ε*	*Z*	*Z* ^w^
0.19	14.8	34	204	702	1670
0.20	15.7	23	115	691	1320
0.28	24.0	8	64	599	1440
0.32	20.3	16	112	602	1640

Since the fixed wall particles underwent extended handling, including prolonged exposure to air, their charge and Yukawa-energy scale is expected to be higher than the ones of the mobile and immobile fluid particles. Thus, the value of the Yukawa-energy scale was separately adjusted for the mobile and immobile fluid particles, *ε*_Y_, and the fixed wall particles, *ε*^w^_Y_ (Table 1).

Based on the values of the inverse Debye length *κ*, the Yukawa-energy scales, *ε*_Y_ and *ε*^w^_Y_, the particle diameters, *σ*_p_ and *σ*^w^_p_, and the Bjerrum length in CHB6, *λ*_B_ = 7 nm, the number of elementary charges of the fluid particles,3
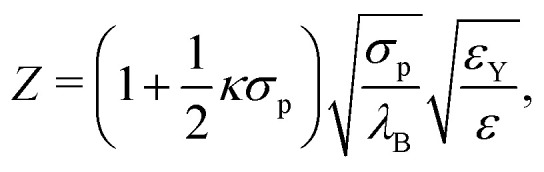
and similarly the number of charges of the fixed wall particles, *Z*^w^, were determined (Table 1). The number of charges of the fluid particles are very similar for the different samples, as expected for particles from the same batch. The number of charges 600 ≲ *Z* ≲ 700 are slightly higher than reported previously (100 ≲ *Z* ≲ 500)^83^ which is also attributed to the sample preparation procedure, especially the exposure to air of a thin layer of sample and hence a relatively large sample surface during the spreading on the cover glass. The exposure to air was much more prolonged for the fixed wall particles. Correspondingly, the number of their charges are about double as high, 1300 ≲ *Z*^w^ ≲ 1700, with also a relatively small variation between the samples.

### 2.3 Fundamental-measure theory and Ornstein–Zernicke equation

Density profiles were calculated using fundamental-measure theory (FMT). They were also used as input for the integral-equation theory of inhomogeneous fluids to determine radial-distribution functions and structure factors.

FMT is similarly applied as previously^84^ but extended to soft walls. It is based on density-functional theory and as such also minimizes the functional for the grand potential. The minimization directly leads to^85,86^4
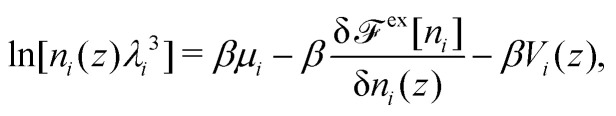
with *β* = 1/*k*_B_*T*, the thermal wavelength *λ*_*i*_, the local number density *n*_*i*_(*z*), the chemical potential *μ*_*i*_,^87^ and the wall potential *V*_*i*_(*z*) of component *i* where the different components are hard-spheres of different sizes *σ*_p,*i*_.^84^ The confining direction, *i.e.* the direction perpendicular to the walls, is denoted by *z* with *z* = 0 in the center of the slit while *x* and *y* indicate the directions parallel to the walls (Fig. 1). The White-Bear Version II^86^ serves as approximation for the excess free energy functional 
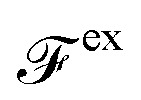
 of a mixture of hard-spheres. To determine the density profiles *n*_*i*_(*z*), eqn (4) is self-consistently solved in an iterative procedure.^86^ In each iteration, the chemical potential *μ*_*i*_ is adapted such that the number distribution of sizes is Gaussian with mean *σ*_p_ and standard deviation *δ*_p_.^84^

The potential *V*_*i*_(*z*) is not a hard wall potential, as in ref. 84, but describes a slit with soft walls, similar but not identical to ref. 88, and is given by5
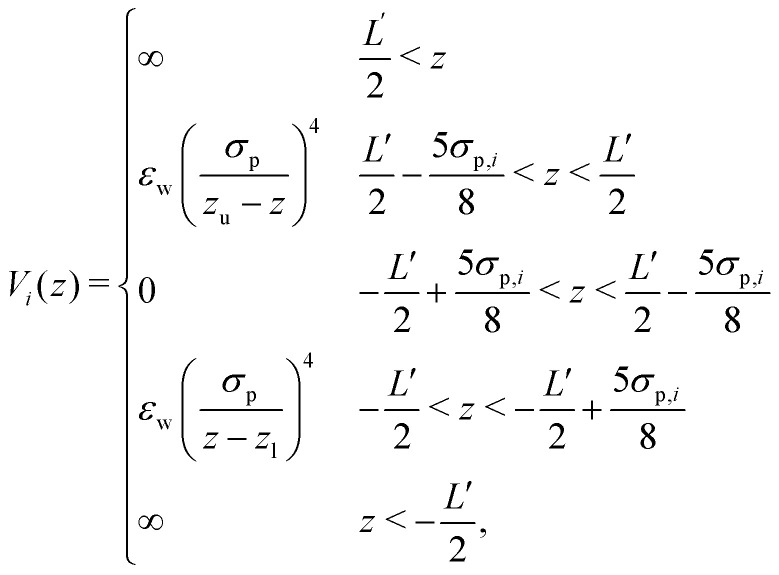
with *ε*_w_ = *ε*, *z*_u_ = *L*′/2 + (5/8)*σ*_p_, *z*_l_ = −*z*_u_, *L*′ = *L* + Δ*L* and Δ*L* = 0.35*σ*_p_ takes into account the softness. The soft potential in the vicinity of the walls can be interpreted as an effective potential representing the rough wall created by the fixed wall particles and immobile fluid particles. The potential and parameters were chosen such that the peak heights and the wall separation *L* in the calculated density profiles roughly correspond to the ones observed in experiments and simulations. Both, *L* in the experiments and simulations as well as *L*′ in the FMT, refer to the width accessible to the particle centers not the whole particles.

Based on the density profile 
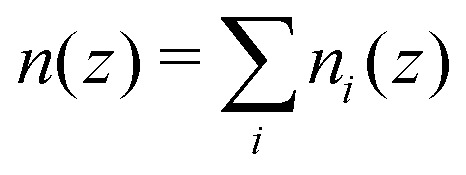
 structural properties can be calculated using a confined version of the Ornstein–Zernicke equation^89^ in combination with a Percus–Yerwick closure. In particular, based on the direct correlation function *h*_*z*_(*r*), the two-dimensional radial distribution function *g*^2D^_*z*_(*r*) = *h*_*z*_(*r*) + 1 (Section 2.4.2) can be calculated. Details of the algorithm are given in ref. 37 and 90. This algorithm requires monodisperse particles and hence, in this step of the theoretical calculation, polydispersity is neglected.

### 2.4 Relevant observables

#### 2.4.1 Density profile

Whereas FMT directly provides the density profile (Section 2.3), based on the experimental and simulation data the number density profile is calculated as6
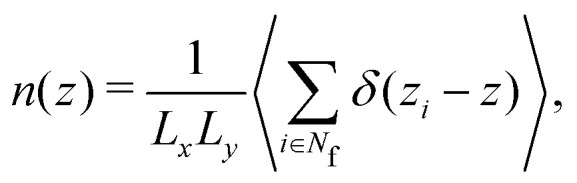
with the position in *z* direction of particle *i* denoted by *z*_*i*_ and the box lengths in *x* and *y* direction by *L*_*x*_ and *L*_*y*_ respectively. The sum considers all fluid particles with their number denoted by *N*_f_ and takes into account their centers rather than their volumes. (Only for the identification of the kind of particle, fixed wall particles and immobile as well as mobile fluid particles, all *N* particles are considered, Section 2.2.2.) Here and in the following the average is an average over time and hence, implicitly, an average over independent configurations. The density profile is evaluated using a bin size *Δ*_*n*_ = 0.054*σ*_p_ = 0.10 μm.

#### 2.4.2 Radial distribution function

Due to the confinement in *z* direction, two-dimensional radial distribution functions (2D-RDFs) in planes parallel to the walls were considered and calculated by7
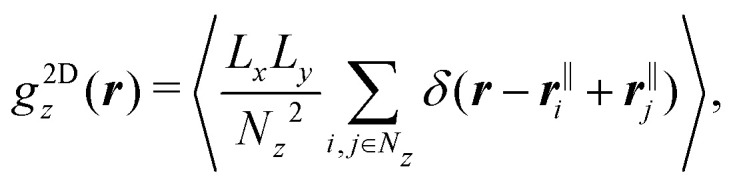
with the position in *x* and *y* direction of particle *i* denoted by ***r***^‖^_*i*_ = (*x*_*i*_,*y*_*i*_). The sum includes the *N*_*z*_ particles in a slab of width *Δ*_*z*_ = 0.97 *σ*_p_ = 1.80 μm centered around height *z*. Typically a height *z*_max_ is chosen which corresponds to a maximum of the density profile *n*(*z*_max_). Considering the isotropy of the system in the directions parallel to the walls, the 2D-RDFs were binned in annuli with inner and outer radii *r* + *kΔ*_*r*_ and *r* + (*k* + 1)*Δ*_*r*_ with *Δ*_*r*_ = 0.070*σ*_p_ = 0.13 μm.

In the case of FMT, the 2D-RDFs were calculated from the direct correlation function *h*_*z*_(*r*) according to *g*^2D^_*z*_(*r*) = *h*_*z*_(*r*) + 1 (Section 2.3).

#### 2.4.3 Structure factor

Two different definitions of a structure factor are considered; the anisotropic and the generalized structure factors.^8,16,18,19,29,30^

The anisotropic structure factor has previously been determined in scattering experiments with confined samples.^8,29,30^ It is defined as8
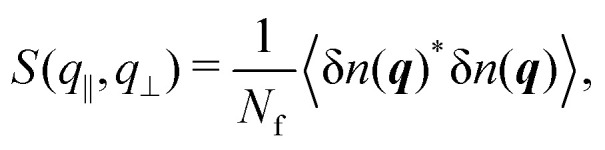
with the wavevector ***q*** = (*q*_*x*_,*q*_*y*_,*q*_*z*_) and its magnitude parallel to the walls, *q*_‖_ = |**q**_‖_| = (*q*_*x*_^2^ + *q*_*y*_^2^)^1/2^, and perpendicular to the walls, *q*_⊥_ = *q*_*z*_. The density mode is defined as9
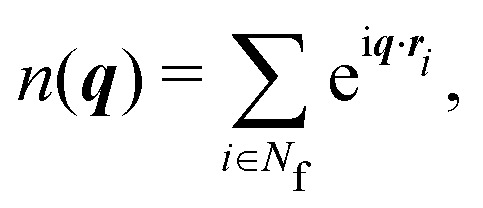
and its fluctuations as δ*n*(***q***) = *n*(***q***) − 〈*n*(***q***)〉.

The generalized structure factor takes into account the broken translational invariance. Thus it is described by two discrete modes10
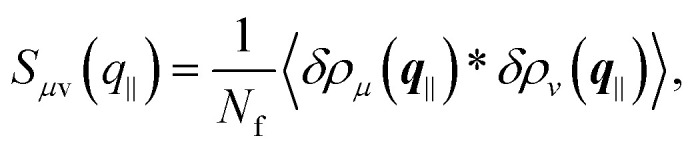
where the density modes are given by11
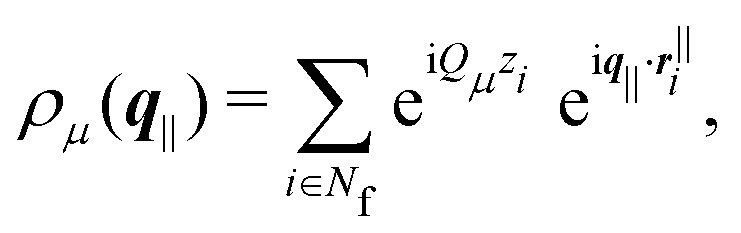
and its fluctuations by δ*ρ*_*μ*_(***q***_‖_) = *ρ*_*μ*_(***q***_‖_) − 〈*ρ*_*μ*_(***q***_‖_)〉 with the wavenumbers *Q*_*μ*_ = 2π*μ*/*L*, 
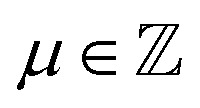
.

Both structure factors are complementary. The anisotropic structure factor *S*(*q*_‖_,*q*_⊥_) is often experimentally easier or solely accessible while the generalized structure factor *S*_*μν*_(*q*_‖_) contains additional information. To illustrate this, the density-density correlation function *G*(*r*^‖^,*z*,*z*′)^91^ is represented in terms of the generalized structure factor *S*_*μν*_(*q*_‖_),12
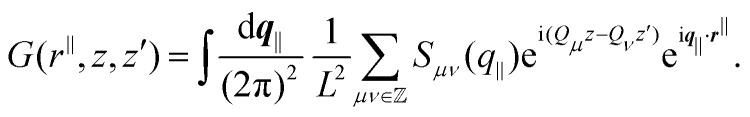
Then the anisotropic structure factor *S*(*q*_‖_,*q*_⊥_),13
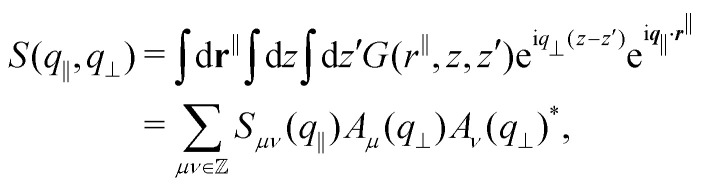
with14
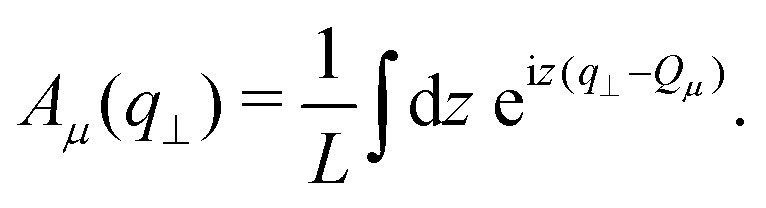
Whereas *S*_*μμ*_(*q*_‖_) = *S*(*q*_‖_,2π*μ*/*L*), the off-diagonal components of the generalized structure factor *S*_*μν*_(*q*_‖_) cannot be recovered from the anisotropic structure factor *S*(*q*_‖_,*q*_⊥_) because the latter only contains information on separations *z*–*z*′. Hence the generalized structure factor *S*_*μν*_(*q*_‖_) contains more information. Nevertheless, the anisotropic structure factor *S*(*q*_‖_,*q*_⊥_) can also reveal information that is not easily accessible through *S*_*μν*_(*q*_‖_), which is exploited in our analysis (Section 3.3).

#### 2.4.4 Volume fraction

The volume fraction *ϕ* is based on the number of mobile fluid particles, *N*_f_, present in the volume *L*_*x*_*L*_*y*_ (*L* + *σ*_p_), *i.e.*15
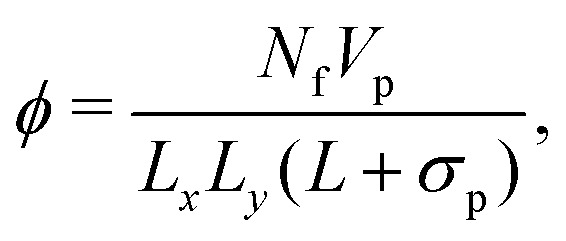
with the particle volume *V*_p_ = π*σ*_p_^3^/6.

## 3 Results and discussion

The effects of confinement on colloidal fluids with moderate volume fractions were investigated. The fluids were confined by rough walls. The roughness is due to particles attached to the walls; ‘fixed wall particles’ and ‘immobile fluid particles’. The immobile fluid particles are identical to the (mobile) fluid particles except that they form part of the rough wall for an extended period of time and hardly move, whereas the fixed wall particles are slightly smaller and carry a higher charge than the fluid particles and are permanently part of the rough wall (Sections 2.1.2 and 2.2.2). The proportion of fixed wall particles and immobile fluid particles as well as their arrangement affect the roughness. Since the coverage and arrangement depend on minute details of the experimental preparation procedure, which are difficult to consistently perform (Section 2.1.2, Fig. 2), the roughness shows moderate variations and hence implicitly depends on the parameters varied in this study, namely the slit width and the volume fraction, although the coverage and arrangement do not directly depend on these parameters.

The slit width is the central parameter in the present study. Because the specific thickness of the monolayer of fixed wall particles and immobile fluid particles depends on the details of the preparation, it is not useful to refer to the separation of the surfaces to which they are attached. Instead, we consider the distance *L* between the outermost peaks in the density profile of the fluid particles, *i.e.* not considering the fixed wall particles and immobile fluid particles, which is the separation of the two layers next to the fixed wall particles and immobile fluid particles at the bottom and at the top of the sample (Fig. 1). In the limit of flat walls, corresponding to uncovered walls and hence an infinitely thin layer of fixed wall particles and immobile fluid particles, this definition corresponds to the previously defined *accessible slit width*.^18,23^ In the following we will refer to *L* as the confinement length. The mentioned values of *L* are nominal values because the experimental and simulation values slightly differ.

In this study we varied the confinement length *L* and the volume fraction *ϕ* and investigated the effects of these parameters on the structure of the confined fluid. The particle arrangement in the slit is quantified by the density profile *n*(*z*), which characterizes the distribution perpendicular to the walls (Fig. 1, Section 3.1), and the two-dimensional radial distribution function *g*^2D^_*z*_(*r*), which characterizes the arrangement parallel to the walls and is determined at wall distances corresponding to maxima in the density profile, *z*_max_ (Section 3.2). In addition, the anisotropic structure factor *S*(*q*_‖_,*q*_⊥_) and the generalized structure factor *S*_*μν*_(*q*_‖_) provide information on the arrangement perpendicular and parallel to the walls (Section 3.3). The discussion of these parameters focuses on the most dilute, *ϕ* = 0.19, and most dense, *ϕ* = 0.32, samples because the results for intermediate volume fractions, *ϕ* = 0.20 and 0.28, lie in between these two cases and do not show qualitatively different behaviour.

### 3.1 Density profiles – layering

Perpendicular to the walls, the confinement leads to density variations. They are quantified by the density profile *n*(*z*) (eqn (6), Fig. 1) which was determined in experiments, simulations, and theory for different confinement lengths *L* and volume fractions *ϕ*. The density profile *n*(*z*) is only based on the mobile fluid particles thus disregarding the layers of fixed wall particles and immobile fluid particles.

**Fig. 3:**
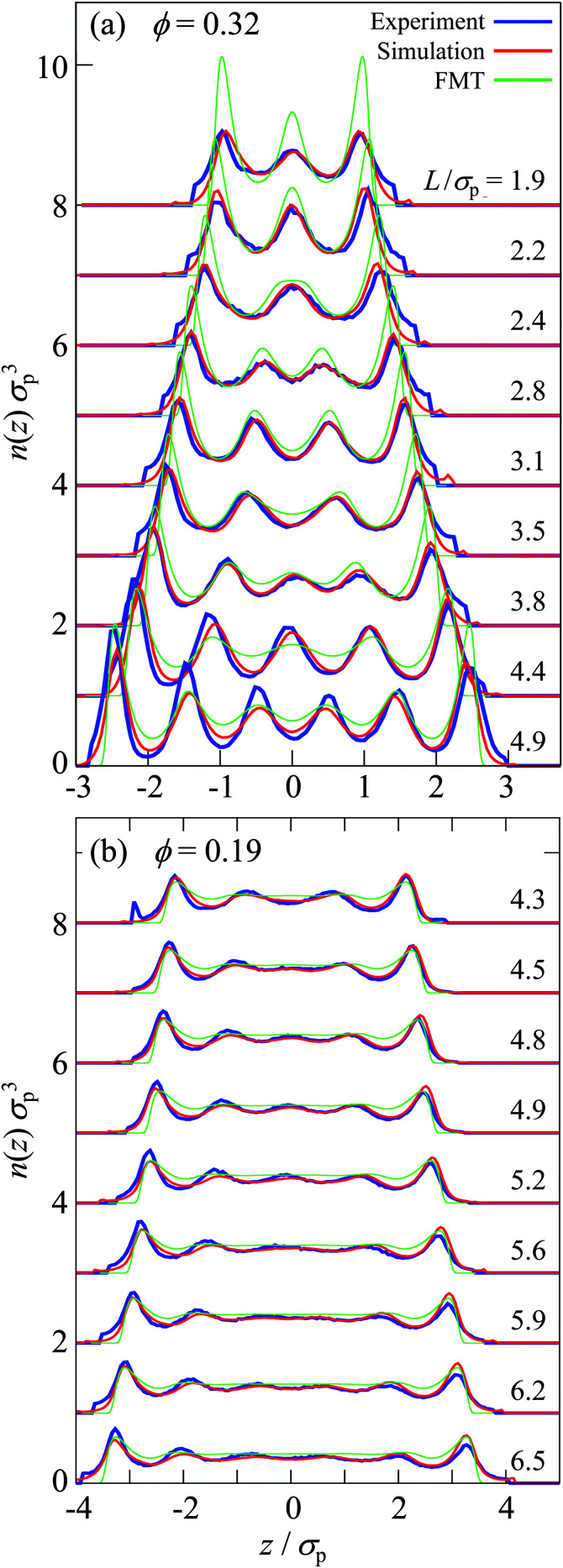
Number density profile *n*(*z*) normalized by the cube of the particle diameter *σ*_p_^3^ for samples with volume fractions (a) *ϕ* = 0.32 and (b) *ϕ* = 0.19 for different confinement lengths *L* (as indicated) as obtained in experiments (blue), simulations (red) and theory (green). The distance to the central plane, *z*, is normalized by the particle diameter *σ*_p_. The profiles are shifted for clarity.

**Fig. 4:**
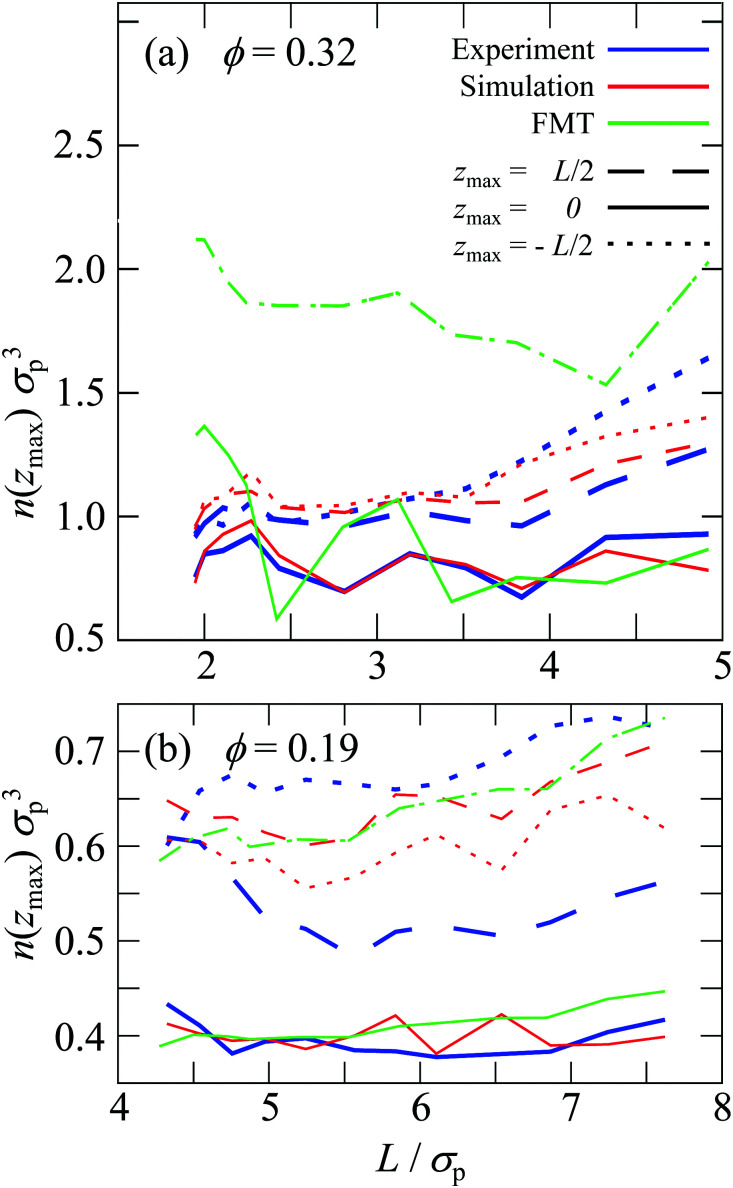
Peak heights of the density profiles *n* (*z*_max_) normalized by the cube of the particle diameter *σ*_p_^3^ for the samples with volume fractions (a) *ϕ* = 0.32 and (b) 0.19 as a function of confinement length *L* normalized by the particle diameter *σ*_p_ as obtained in experiments (blue), simulations (red) and theory (green). Shown are the height of the central peak at *z*_max_ ≈ 0 (solid lines), the height of the peak close to the upper wall at *z*_max_ = *L*/2 (dashed lines) and the height of the peak close to the lower wall at *z*_max_ = −*L*/2 (dotted lines).

The sample with the highest volume fraction *ϕ* = 0.32 exhibits pronounced density variations (Fig. 3a). For the smallest confinement length *L* ≈ 2*σ*_p_, the density profile *n*(*z*) shows three peaks. With increasing confinement length *L*, the central peak becomes more pronounced, then decreases and broadens to subsequently split into two distinct peaks. This sequence of increase, decrease and splitting of the central peak(s) is repeated upon further increasing the confinement length *L*. This is reflected in the height of the central peak or, in the case of two central peaks, the peak with *z* ≳ 0. It shows a non-monotonic dependence on the confinement length *L* (Fig. 4a, solid lines). The period of these oscillations is approximately one particle diameter *σ*_p_. In contrast, the heights of the outermost peaks steadily increase with confinement length *L* (Fig. 4a, dashed and dotted lines) which might also be caused by accidental variations in the wall coverage (Fig. 2), as discussed below (Section 3.3.1). The sample with the lowest volume fraction, *ϕ* = 0.19, shows similar density variations which, however, are significantly less pronounced with respect to the amplitude of the modulations in *n*(*z*) as well as the non-monotonic dependence of the height of the central peak and the increase of the height of the outermost peaks (Fig. 3b and 4b).

The density profiles determined in experiments and simulations quantitatively agree, despite the complex boundaries and interaction potential. For all data sets of each sample, *i.e.* all confinement lengths *L*, only three adjustable parameters are involved, namely the inverse Debye length *κ*, the Yukawa-energy scale of the fluid particles, *ε*_Y_, and of the fixed wall particles, *ε*^w^_Y_ (Section 2.2.3 with Table 1). Small deviations are observed for the outermost peaks, which are attributed to the empirical description and identification of the fixed wall particles and immobile fluid particles. In addition, there is a small bump in the experimental density profile for *ϕ* = 0.19 and *L*/*σ*_p_ = 4.3 at *z*/*σ*_p_ ≈ −3 which is due to misidentified immobile particles just outside the chosen range for immobile particles, ranging from *L*_w_ to *L*_im_ (Section 2.2.2). At large confinement length *L* and particularly for the largest volume fraction, *ϕ* = 0.32, also the other peaks show some differences between experiments and simulations. The simulations yield more symmetric density profiles whereas the experiments tend to show higher peaks at the bottom of the slit than at the top of the slit (compare the dashed and dotted lines in Fig. 4). This is attributed to moderate sedimentation during the experiments which has a stronger effect in thicker samples, *i.e.* larger *L*. While the tendency for sedimentation increases with particle size, a fractionation by particle size was not observed. Although the samples were carefully density matched (Section 2.1.1), a small density mismatch is sufficient to cause noticeable sedimentation due to the large particle size. For example, evaporation during the sample preparation might have caused not only a slight increase in volume fraction (which is taken into account, see Section 2.4.4) but, together with different evaporation rates of *cis*-decalin and CHB6, can change the composition of the solvent mixture and hence its density. In addition, due to different thermal expansion coefficients of the particles and the solvent mixture, small temperature variations might have led to minute density differences between the particles and the solvent mixture.

The density profile was also studied using fundamental-measure theory (FMT). It is based on a simplified soft wall potential and hard-sphere particle–particle interactions, thus disregarding the roughness of the wall and the electrostatic interactions (Section 2.3). Quantitative agreement is therefore not expected. Nevertheless, the behaviour qualitatively agrees with the experimental and simulation findings (Fig. 3) as well as previous theoretical and experimental results.^6,27,29,33–35,45,58^ For the highest volume fraction, *ϕ* = 0.32, strong modulations of the density profiles are observed which qualitatively agree with the experimental and simulation results (Fig. 3a) and are consistent with observations in previous studies comparing hard-spheres and charged spheres.^33–35,45^ However, for the lowest volume fraction, *ϕ* = 0.19, only moderate modulations of the density profiles are predicted (Fig. 3b). They are weaker than observed in experiments and simulations, which is attributed to the missing electrostatic interactions. Furthermore, the height of the central peak as determined by FMT shows variations with the confinement length *L*. The period of the variations is also approximately a particle diameter *σ*_p_ but the amplitude of the variations is more pronounced (Fig. 4a, green solid line).

### 3.2 Radial distribution function – packing

Having analyzed the density profiles describing the layering, we now turn to the packing within the layers. The packing is quantified by the two-dimensional radial distribution function (2D-RDF) *g*^2D^_*z*_(*r*) in planes parallel to the walls and at different positions within the slit, *z*, where the packing around the maxima in the density profile are of particular interest (eqn (7), Fig. 1). The 2D-RDF was determined based on confocal microscopy images. Confocal microscopy provides the necessary spatial resolution in *z* direction. This allows to identify the layers parallel to the walls and avoids averaging in *z* direction and hence mixing the different layers.

**Fig. 5:**
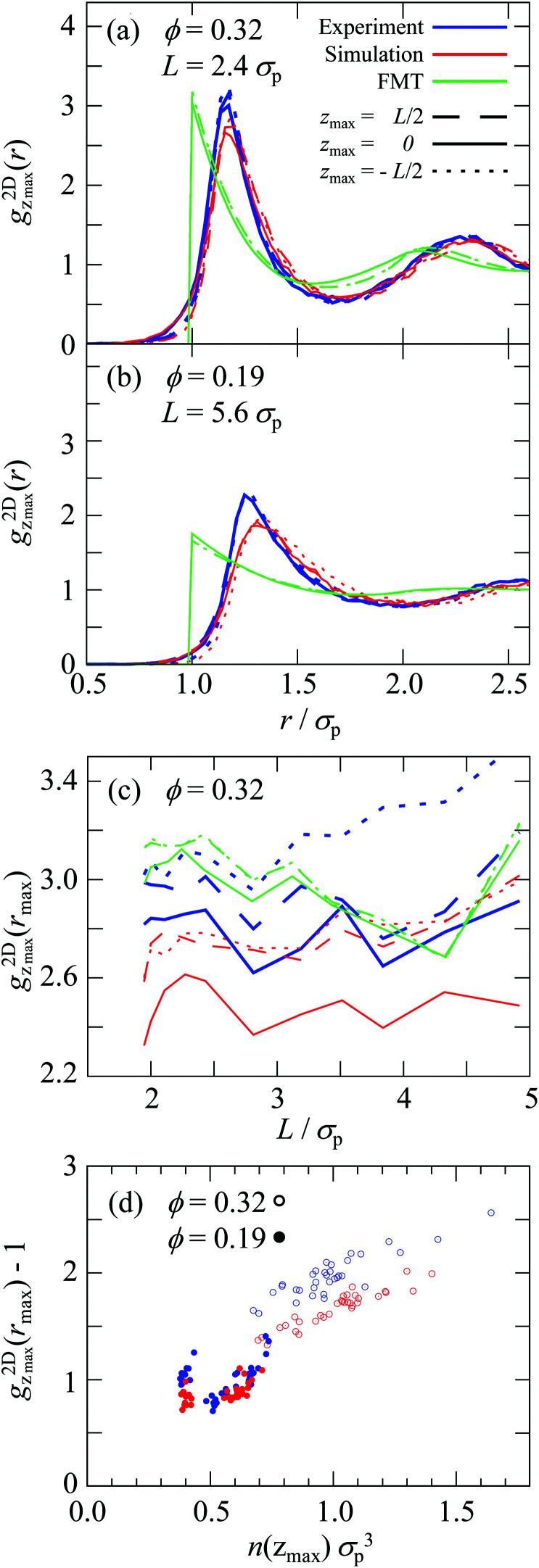
(a and b) Two-dimensional radial distribution function (2D-RDF) 
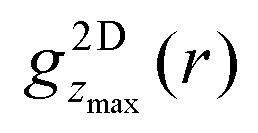
 as a function of the particle separation *r* normalized by the particle diameter *σ*_p_ for volume fractions *ϕ*, confinement lengths *L* and positions within slabs around the central (*z*_max_ = 0), upper (*z*_max_ = *L*/2) and lower (*z*_max_ = −*L*/2) peaks in the density profile *n*(*r*), as indicated. (c) Height of the first maximum, 
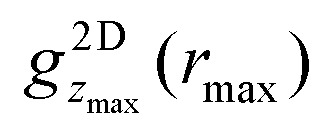
, as a function of the normalized confinement length *L* for different positions *z*_max_ as indicated. (d) Height of the first maximum, 
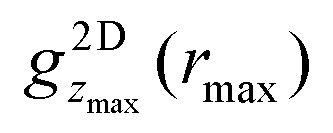
, as a function of the local density *n*(*z*_max_) for data including all studied confinement lengths *L* and positions *z*_max_. Data from experiments (blue), simulations (red) and theory (green).

For all volume fractions *ϕ*, confinement lengths *L* and positions *z*, the *g*^2D^_*z*_(*r*) are qualitatively similar and indicate well-developed shells. Representative *g*^2D^_*z*_(*r*) are shown in Fig. 5a and b. The *g*^2D^_*z*_(*r*) determined by experiments and simulations are very similar with only small but significant differences. In particular, in the simulations the first peak is slightly lower and broader, especially for the less dense sample (Fig. 5a and b). This indicates a slightly less pronounced order but a very similar number of particles in the first shell, variations are found to be smaller than 3%. This is most likely due to the modelling of the electrostatic interactions (Section 2.2.1), consistent with the stronger discrepancy in the less dense sample in which electrostatic interactions are more important.

For the highest volume fraction, *ϕ* = 0.32, the first maximum of 
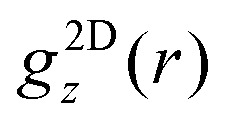
, that is 
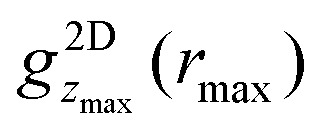
, is smaller in the center of the samples, *z*_max_ = 0, than next to the walls, *z*_max_ = ±*L*/2, (Fig. 5a and c) whereas for the lowest volume fraction, *ϕ* = 0.19, no significant dependence on the position *z* has been observed beyond the expected statistical uncertainty (Fig. 5b). Also for *ϕ* = 0.32 the differences are small but significant and observed for all confinement lengths *L* in experiments and simulations. Furthermore, for all positions *z*, the height of the first maximum shows modulations with a period of approximately one particle diameter *σ*_p_. Although these modulations are clearly visible, their amplitude is relatively small. The difference between the central, *z* = 0, and outermost, *z* = ±*L*/2, layers as well as the non-monotonic behavior as a function of the confinement length *L* resemble the behaviour of the density profiles (Fig. 4a). This indicates that the packing strongly depends on the local density.

To investigate the relation between the packing and local density, the correlation between 
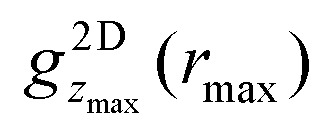
 and *n*(*z*_max_) is investigated. A correlation is indeed found (Fig. 5d). Irrespective of the volume fraction *ϕ*, confinement length *L* and position *z*_max_, a general dependence of the packing, quantified by 
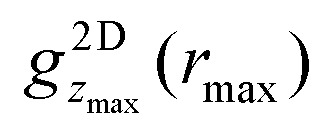
, and the local density, quantified by *n*(*z*_max_), is observed, except for the above-mentioned slightly lower (and broader) peak in the simulations.

The FMT allows for a direct evaluation of *g*^2D^_*z*_(*r*) based on the direct correlation function (Section 2.3). Since only hard-sphere particle–particle interactions and a flat soft wall instead of a rough wall are considered, no quantitative agreement with the results from experiments and simulations is expected. In particular, the pure hard-sphere interactions lead to the jump of *g*^2D^_*z*_(*r*) at contact, *i.e. r* = *σ*_p_. The *g*^2D^_*z*_(*r*) of FMT is closer to the experimental and simulation findings for the sample with the high volume fraction, *ϕ* = 0.32, in which hard-sphere interactions dominate, than the less dense sample, *ϕ* = 0.19, in which electrostatic interactions are more important. This particularly concerns the height of the first maximum and the relative heights in the central and outermost layers, although the difference between the central and outermost layers is much smaller (Fig. 5a–c). The slightly stronger packing in the central compared to the outermost layers predicted for the low volume fraction (Fig. 5b) is due to the fact that, at the positions of the outermost layers, the direct correlation function has already considerably decayed due to the rapid decay of the density to zero (Fig. 3b), and therefore is a technical artifact and also prohibits a reliable determination of 
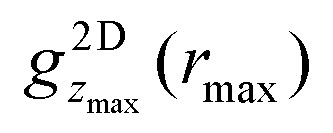
 as a function of *n*(*z*_max_) (Fig. 5d).

### 3.3 Structure factors – layering and packing

All experimental results were obtained using imaging and hence in real space. Nevertheless, in the following we will consider the effects of confinement on the particle arrangement in terms of the structure factor, which characterizes the arrangement in wavevector space and provides an average across the slit. It is usually determined in scattering experiments. Typically the anisotropic structure factor *S*(*q*_‖_,*q*_⊥_) is obtained. In addition, we calculate the generalized structure factor *S*_*μν*_(*q*_‖_) (Section 2.4.3). The structure factor enables a direct comparison with results from scattering experiments and highlights aspects of the packing that are less obvious in real space. As for the density profile *n*(*r*) and the 2D-RDF *g*^2D^_*z*_(*r*), only the *N*_f_ mobile fluid particles are taken into account whereas the fixed wall particles and the immobile fluid particles are not considered (Section 2.4).

#### 3.3.1 Anisotropic structure factor

**Fig. 6:**
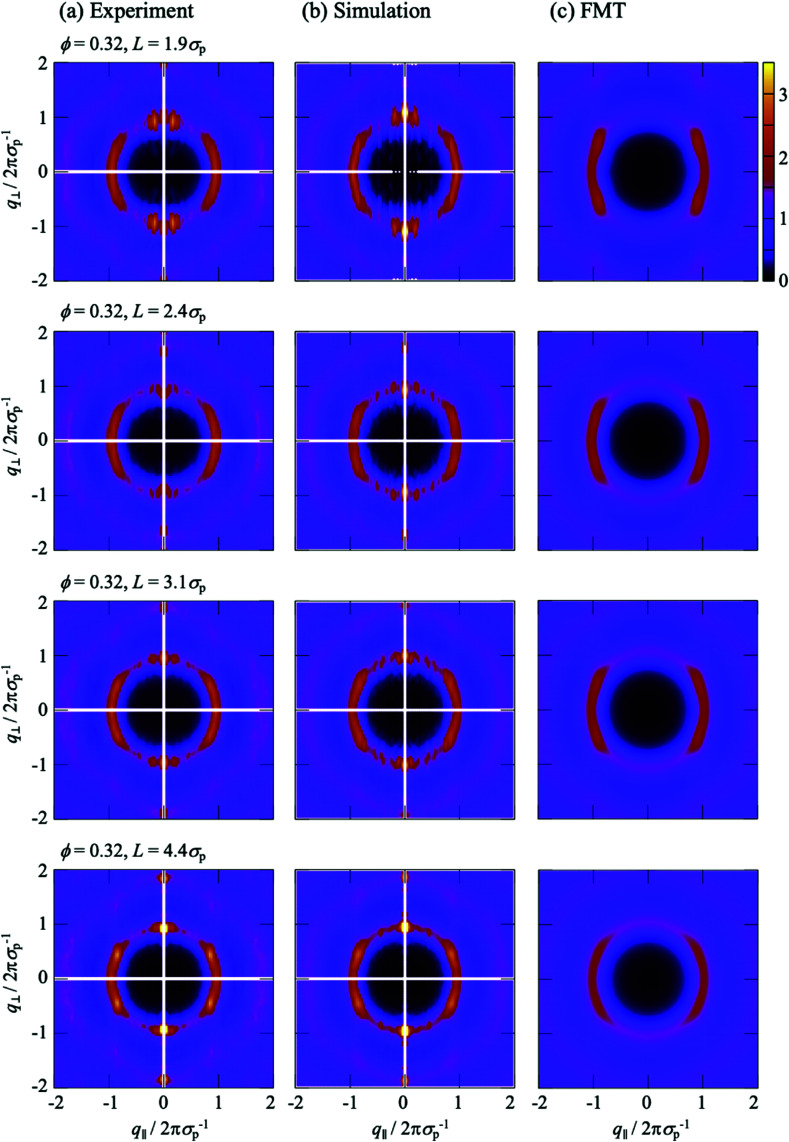
Anisotropic structure factor *S*(*q*_‖_,*q*_⊥_) as a function of the magnitudes of the wavevector components in parallel, *q*_‖_, and perpendicular, *q*_⊥_, direction to the walls normalized by the particle diameter *σ*_p_ for different confinement lengths *L* (as indicated) as obtained in (a) experiments, (b) simulations and (c) theory. The volume fraction *ϕ* = 0.32.

**Fig. 7:**
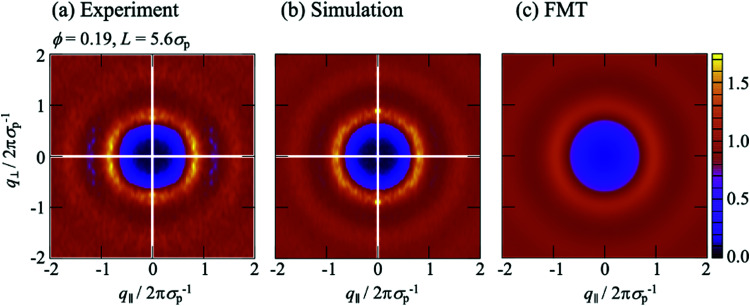
Anisotropic structure factor *S*(*q*_‖_,*q*_⊥_) as a function of the magnitudes of the wavevector components in parallel, *q*_‖_, and perpendicular, *q*_⊥_, direction to the walls normalized by the particle diameter *σ*_p_ for confinement length *L* = 5.57*σ*_p_ as obtained in (a) experiments, (b) simulations and (c) theory. The volume fraction *ϕ* = 0.19.

The anisotropic structure factor *S*(*q*_‖_,*q*_⊥_) was calculated for different confinement lengths *L* and volume fractions *ϕ*. We start with the theoretical results which correspond to a more basic situation; hard-sphere particle–particle interactions and flat soft walls (Fig. 6c and 7c). For small confinement lengths *L*, the structure factor *S*(*q*_‖_,*q*_⊥_) shows two extended peaks for (*q*_‖_,*q*_⊥_) ≈ (±2π*σ*_p_^−1^,0) which implies pronounced packing parallel to the walls with correlations and hence some order within the layers. This is consistent with the well-developed and essentially *z* independent shell structure indicated by *g*^2D^_*z*_(*r*) (Fig. 5a–c), where the *z* independence ensures that peaks are not washed out despite the averaging over the position *z*. Perpendicular to the walls, *S*(*q*_‖_,*q*_⊥_) exhibits maxima at *q*_⊥_ = 2π*σ*_p_^−1^ and 4π*σ*_p_^−1^ but they are less pronounced indicating weak correlations between the layers. The anisotropy in *S*(*q*_‖_,*q*_⊥_) decreases upon increasing the confinement length *L* or decreasing the volume fraction *ϕ* due to the increased fraction of particles located in the less ordered center of the slit (Fig. 3). These observations are in agreement with previous findings,^6,29,30^ except that no significant modulations in *S*(*q*_‖_,*q*_⊥_) are observed upon changing the confinement length *L* by *σ*_p_ (Fig. 6c, sequence *L*/*σ*_p_ = 1.95, 2.43, 3.10), although such modulations are observed in the density profile *n*(*z*) and 2D-RDF *g*^2D^_*z*_(*r*) (Fig. 4). This is attributed to the relatively large confinement lengths *L* ≳ 2*σ*_p_ and the flat soft walls which further reduce these modulations.

The experimental and simulation results quantitatively agree with each other (Fig. 6a, b and 7a, b). They, however, refer to a more complex situation than considered with FMT due to the combination of hard-sphere and electrostatic interactions and especially due to the roughness of the walls. Nevertheless, there are several similarities. Most prominent, the *S*(*q*_‖_,*q*_⊥_) from experiments and simulations also show two extended peaks for (*q*_‖_,*q*_⊥_) ≈ (±2π*σ*_p_^−1^,0) indicating pronounced order parallel to the walls. With increasing confinement length *L* and decreasing volume fraction *ϕ*, the two peaks evolve towards modulated rings, similar to previous observations in a similar system.^29^ This corresponds to the pronounced and *z* independent shell structure indicated by the 2D-RDF *g*^2D^_*z*_(*r*) (Fig. 5a–c), as described above for the FMT data.

The *S*(*q*_‖_,*q*_⊥_) from experiments and simulations also show modulations perpendicular to the walls at *q*_⊥_ = 2π*σ*_p_^−1^ and 4π*σ*_p_^−1^, similar to the *S*(*q*_‖_,*q*_⊥_) from FMT and in agreement with the density profiles *n*(*z*) (Fig. 3). There are, however, also additional features. Pronounced peaks are observed at (*q*_‖_,*q*_⊥_) ≈ (0,±2π*σ*_p_^−1^) for all confinement lengths *L* and, although with different intensities, also for all volume fractions *ϕ*. Further peaks at (0,±4π*σ*_p_^−1^) are visible at least for the highest volume fraction *ϕ* = 0.32. For *L* = 1.95*σ*_p_ and less so for *L* = 2.43*σ*_p_, these peaks at very small *q*_‖_≈0 are accompanied by peaks at larger but still small *q*_‖_, approximately (±0.2 × 2π*σ*_p_^−1^,±2π*σ*_p_^−1^). These peaks at very small and small *q*_‖_ have not been reported before, possibly because the diffraction from the confining walls masks them in scattering experiments or because practically flat walls were investigated.^6,29^ Since these peaks occur in experiments and simulations with rough walls (Fig. 6a, b and 7a, b) but not in the presence of flat walls (Fig. 6c and 7c), they are believed to be due to the roughness of the walls.

**Fig. 8:**
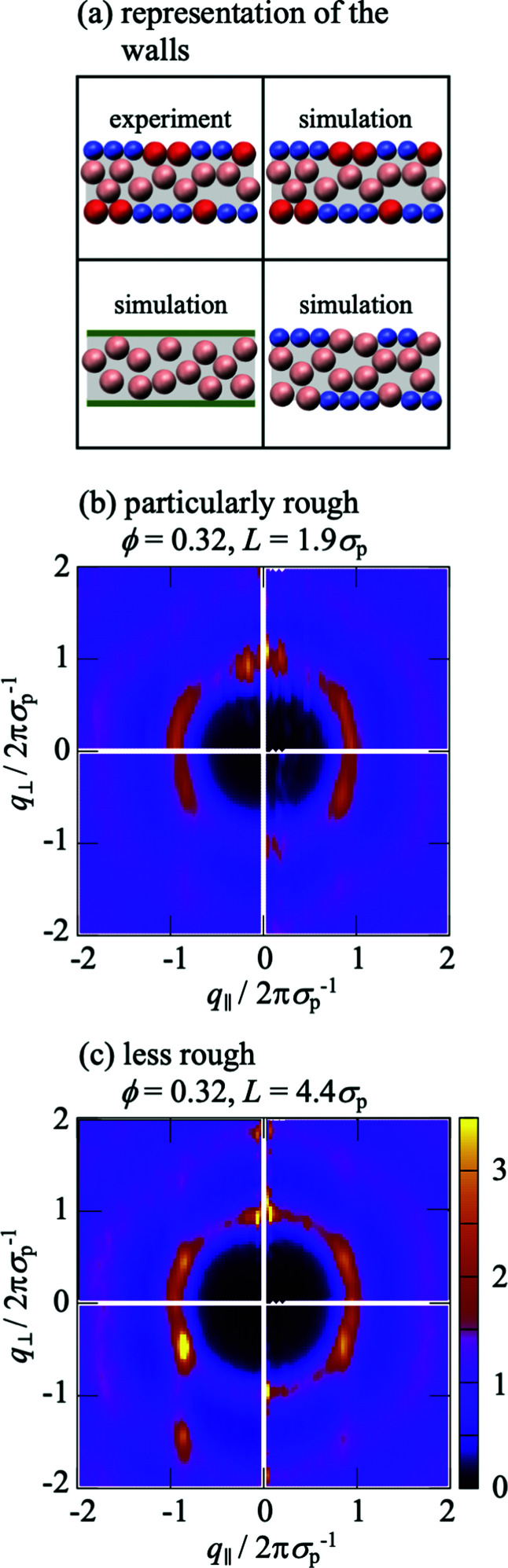
Anisotropic structure factor *S*(*q*_‖_,*q*_⊥_) as a function of the magnitudes of the wavevector components in parallel, *q*_‖_, and perpendicular, *q*_⊥_, direction to the walls normalized by the particle diameter *σ*_p_. As indicated in (a), experimental data are shown in the top left quadrant, whereas the other quadrants show simulation data. The top right quadrant shows the results of the simulations that mimic the experimental situation with fluid particles (pink) confined by fixed wall particles (blue) and immobile fluid particles (red); the bottom right quadrant shows the results of the simulations that omit the immobile fluid particles and hence the fluid particles are confined by fixed wall particles and the gaps are filled by mobile fluid particles; the bottom left quadrant shows the results of simulations omitting the immobile fluid particles and the fixed wall particles and hence the fluid particles are confined by flat hard walls (green); The walls in the experiments are (b) particularly rough, *i.e.* heterogeneous due to a large fraction of immobile fluid particles (Fig. 2, left), and (c) less rough, *i.e.* more homogeneous due to a small fraction of immobile fluid particles (Fig. 2, right). The volume fraction *ϕ* = 0.32.

To investigate the effects of the wall roughness, *S*(*q*_‖_,*q*_⊥_) was determined in simulations for the experimental walls as well as increasingly modified walls (Fig. 8a). In the experiments, the walls are formed by fixed wall particles and immobile fluid particles (Section 2.2.2). The roughness, especially its range of length scales, depends on the ratio of fixed wall particles to immobile fluid particles since their sizes and charges are different (Section 2.2.3 with Table 1). Two cases are investigated; a similar fraction of both species and hence a particularly rough wall with a roughness on a broad range of length scales (Fig. 2, left; Fig. 8b) and mainly fixed wall particles and hence a more homogeneous rough wall with a roughness on a limited range of length scales (Fig. 2, right; Fig. 8c). (Note that the link between the roughness and the confinement length *L* is accidental.) The experimental findings (Fig. 8b and c, top left quadrant; identical to Fig. 6a, top and bottom respectively; note that all four quadrants in Fig. 6 refer to the same conditions) are well reproduced by the simulations (Fig. 8b and c, top right quadrant; identical to Fig. 6b, top and bottom respectively), as already discussed in the context of Fig. 6a and b. First the particularly rough wall is modified (Fig. 8b). The fixed wall particles are retained but the immobile fluid particles are replaced by mobile fluid particles (Fig. 8b, bottom right quadrant). The pronounced peak at very small *q*_‖_ is now weaker and the peak at small *q*_‖_ disappeared. This indicates that the roughness-related order on the smaller length scale, which still corresponds to a few particle sizes, is reduced while the order on a larger length scale is essentially unaffected. This is attributed to the replacement of immobile fluid particles that represent a fixed roughness by mobile fluid particles that result in a time-averaged, reduced roughness. In addition, the heterogeneity on a larger length scale, caused by the presence of fixed wall particles and mobile fluid particles, essentially remains. This is consistent with the case of the less rough walls (Fig. 8c); only a few immobile fluid particles are present and, correspondingly, no pronounced peak at small *q*_‖_ is observed whereas a pronounced peak is detected at very small *q*_‖_. In addition, the better agreement of the experimental data with the simulation data obtained in the presence of immobile particles indicates that describing the fluid particles in the uncovered regions as immobile is justified (Section 2.2.2).

If the fixed wall particles are successively removed, the peaks at small and very small *q*_‖_ gradually vanish in the case of both, the particularly rough and less rough wall (data not shown). Once all fixed wall particles are replaced by a flat hard wall (Fig. 8a, bottom left quadrant), all peaks at small and very small *q*_‖_ vanish and the obtained *S*(*q*_‖_,*q*_⊥_) (Fig. 8b and c, bottom left quadrant) resembles the one theoretically predicted for a flat wall (Fig. 6c). In summary, these observations indicate that the roughness of the wall is indeed responsible for the arrangement of the mobile fluid particles that results in the additional peaks. The peak at small *q*_‖_ seems to reflect the particle arrangement induced by the roughness due to the immobile fluid particles. In contrast, the peak at very small *q*_‖_ is mainly due to the longer-range heterogeneity of the fixed wall particles. Moreover, a comparison of the effects of a particularly rough and a less rough wall (Fig. 8b and c) reveals that the roughness mainly affects the ordering in the layers parallel to the walls, *i.e.* the dependence of *S*(*q*_‖_,*q*_⊥_) on *q*_‖_, whereas the layering perpendicular to the walls is only affected by the details of the roughness if the roughness is only moderate. These results also illustrate that a faithful representation of the experimental wall in the simulations requires to carefully take into account the fixed wall particles as well as the immobile fluid particles.

#### 3.3.2 Generalized structure factor

**Fig. 9:**
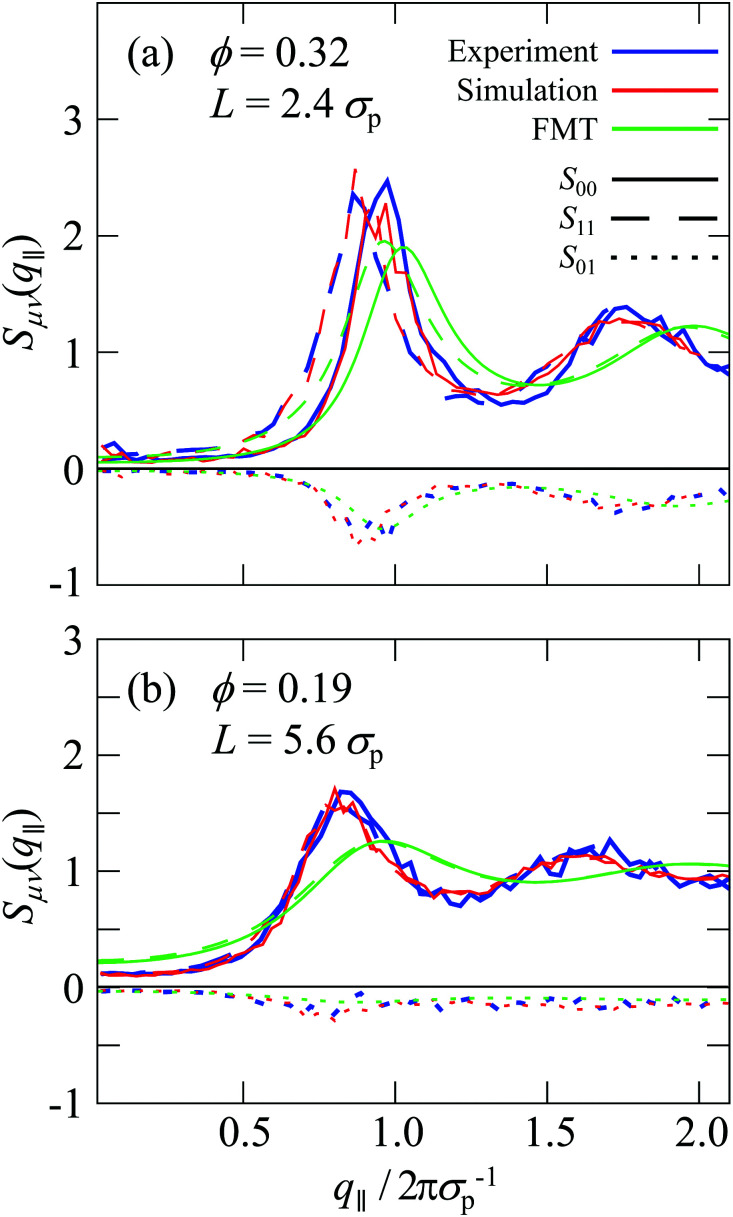
Generalized structure factor *S*_*μν*_(*q*_‖_) as a function of the magnitude of the wavevector component in parallel direction to the walls, *q*_‖_, normalized by the particle diameter *σ*_p_, as obtained in experiments (blue), simulations (red) and theory (green). Shown are three components, *S*_00_ (solid line), *S*_11_ (dashed line) and *S*_01_ (dotted line). The volume fraction *ϕ* and confinement length *L* are indicated.

The diagonal elements of the generalized structure factor, *S*_*μμ*_(*q*_‖_), correspond to cuts of the two-dimensional anisotropic structure factor *S*(*q*_‖_,*q*_⊥_) with constant *q*_⊥_ (Section 2.4.3). They hence contain information that has been discussed in the previous section. The first two diagonal elements, *S*_00_(*q*_‖_) and *S*_11_(*q*_‖_), are illustrated for two different confinement lengths *L* and volume fractions *ϕ* in Fig. 9 and are qualitatively comparable to the *S*_00_(*q*_‖_) of denser fluids confined by flat walls.^19,20^ Experiments and simulations yield very similar *S*_*μμ*_(*q*_‖_). In contrast, the peaks of the *S*_*μμ*_(*q*_‖_) obtained using FMT are shifted to slightly larger *q*_‖_. This is attributed to the missing repulsive Yukawa potential which results in a smaller effective particle size. Compared to *S*_00_(*q*_‖_), the next diagonal element *S*_11_(*q*_‖_) exhibits peaks at smaller values of *q*_‖_. This is consistent with the anisotropic structure factor *S*(*q*_‖_,*q*_⊥_) (Fig. 6 and 7) which shows arc-like peaks that hence are located at *q*_‖_ = ((2π*σ*_p_^−1^)^2^ − *q*_⊥_^2^)^1/2^. Therefore, with increasing *q*_⊥_ the location of the peak shifts to smaller *q*_‖_.

Beyond the diagonal element, the generalized structure factor *S*_*μν*_(*q*_‖_) also reflects the broken translational invariance perpendicular to the walls, which is not reflected in the anisotropic structure factor *S*(*q*_‖_,*q*_⊥_). The lowest mode that highlights this effect is *S*_01_(*q*_‖_) which is given by (eqn (10) and (11))16
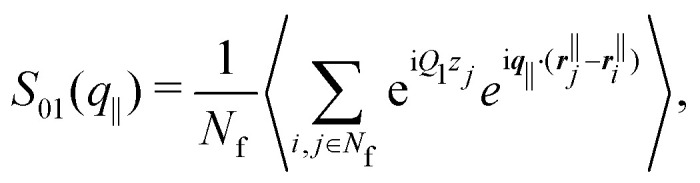
with the wavenumber *Q*_1_ = 2π/*L*. In a translationally invariant system, the term containing *z*_*j*_ would vanish and hence *S*_01_(*q*_‖_) = 0. The inhomogeneous density profile *n*(*z*) of a confined system, however, results in a finite contribution which turns out to be negative (Fig. 9). For small confinement lengths *L* and large volume fractions *ϕ*, significant inhomogeneities in the density profile are present (Fig. 3a) and are reflected in *S*_01_(*q*_‖_) which is significantly different from zero (Fig. 9a). In contrast, for the lowest volume fraction *ϕ* = 0.19 with moderate variations in the density profile *n*(*z*), the values of *S*_01_(*q*_‖_) are considerably smaller (Fig. 9b). These features of the off-diagonal elements are observed for experiments and simulations, which quantitatively agree. The theoretical predictions show moderate differences which are due to the reasons already mentioned, namely, for the higher volume fraction *ϕ* = 0.32 (Fig. 9a), a shift of the maximum to slightly larger *q*_‖_ due to the missing electrostatic interactions and hence an effectively smaller size and, for the lower volume fraction *ϕ* = 0.19 (Fig. 9b), very small variations in the density profile *n*(*z*) (Fig. 3b).

## 4 Conclusions

The structure of colloidal fluids confined between rough walls has been investigated. The colloids were imaged using confocal microscopy and the experimental situation mimicked in computer simulations. The results from experiments and simulations were compared to predictions based on fundamental-measure theory for a more basic situation, namely hard-spheres confined between flat soft walls. For a detailed description of the fluid structure, several structural parameters were determined that describe the layering and packing. Specifically, the layering is characterized by the density profile perpendicular to the walls, *n*(*z*), the packing within these layers is quantified by the two-dimensional radial distribution function *g*^2D^_*z*_(*r*) in layers parallel to the walls and both is contained in the anisotropic, *S*(*q*_‖_,*q*_⊥_), as well as the generalized, *S*_*μν*_(*q*_‖_), structure factors. Although an imaging technique was used and hence real-space information was obtained, the structure factors were determined to achieve a more detailed characterization of the fluid structure but also to allow for a better comparison to previous experimental, simulation and theoretical work.

The structure of the fluid is dominated by excluded volume effects and hence the hard-sphere particle–particle interactions. However, charges are found to significantly contribute in the lower-volume-fraction samples, where excluded volume effects are less prominent. Electrostatic interactions particularly affect the packing whereas the layering is less affected. This is indicated by the agreement between theory, which neglects electrostatic interactions, and experiments as well as simulations, which is better for the density profile *n*(*z*) than the two-dimensional radial distribution function *g*^2D^_*z*_(*r*).

The structure of the confined fluid was shown to strongly depend on the details of the confining walls. Compared to flat walls, rough walls lead to distinct peaks in the structure factor and hence a more pronounced arrangement. By modifying the walls and their roughness, the peaks could be related to the properties of the rough wall. The properties of the walls could hence be linked to the fluid structure that they impose. The strong and complex effects of the wall furthermore imply that it is crucial to accurately mimic the experimental situation in simulations. The agreement between experiments and simulations provides confidence in the procedure applied in our simulations. The developed experimental protocol and computer model can now be exploited to investigate the dynamics of confined fluids, including denser systems.

## Author contributions

T. F. and S. U. E. conceived the project. A. V. B. performed and analyzed the experiments and provided input to the simulations, A. B. Z. B. contributed to the sample preparation and experimental realization, and G. J. performed and analyzed the simulations and FMT calculations. All authors contributed to the interpretation of the data and the writing of the manuscript.

## Conflicts of interest

There are no conflicts to declare.

## Supplementary Material
